# Prospective Acceptability of Digital Therapy for Major Depressive Disorder in France: Multicentric Real-Life Study

**DOI:** 10.2196/53204

**Published:** 2024-05-20

**Authors:** Odile Amiot, Anne Sauvaget, Isabelle Alamome, Samuel Bulteau, Thomas Charpeaud, Anne-Hélène Clair, Philippe Courtet, Dominique Drapier, Emmanuel Haffen, Eric Fakra, Christian Gaudeau-Bosma, Adeline Gaillard, Stéphane Mouchabac, Fanny Pineau, Véronique Narboni, Anne Duburcq, Laurent Lecardeur

**Affiliations:** 1 Groupe Hospitalier Paul Guiraud Boulogne Billancourt France; 2 Movement - Interactions - Performance Centre Hospitalier Universitaire Nantes Nantes Université Nantes France; 3 Centre Hospitalier ESQUIROL Limoges France; 4 Institut national de la santé et de la recherche médicale 1246, MethodS in Patient-Centered Outcomes and HEalth ResEarch Centre Hospitalier Universitaire de Nantes, Department of Addictology, Psychiatry and Old Age Psychiatry University of Nantes Nantes France; 5 Department of Addictology, Psychiatry and Old Age Psychiatry Centre Hospitalier Universitaire de Nantes Nantes France; 6 Clinique du Grand Pré Durtol France; 7 Sorbonne Université Paris France; 8 Neuropsychiatrie: Recherche Epidemiologique et Clinique, Institut national de la sante et de la recherche medicale Centre Hospitalier Universitaire de Montpellier University of Montpellier Montpellier France; 9 Department of Emergency Psychiatry and Acute Care Lapeyronie Hospital Centre Hospitalier Universitaire de Montpellier Montpellier France; 10 Centre Hospitalier Guillaume Régnier Pôle hospitalo universitaire de psychiatrie adulte Rennes France; 11 Centre d’Investigation Clinique Institut national de la sante et de la recherche medicale 1414 équipe neuropsychiatrie du développement et du comportement Université de Rennes Rennes France; 12 Service de Psychiatrie de l’Adulte, Centre d’Investigation Clinique 1431-Institut national de la sante et de la recherche medicale Centre Hospitalier Universitaire de Besançon Université de Franche-Comté Besançon France; 13 Pôle Universitaire de Psychiatrie, Centre Hospitalier Universitaire de Saint-Etienne, Université Jean Monnet, Equipe Troubles psychiatriques, Recherche en Neurosciences et Recherche Clinique- InInstitut national de la sante et de la recherche medicaleserm Centre National de la Recherche Scientifique 5292, Université Lyon 1 Université Jean Monnet Saint-Etienne France; 14 Espace Territoriale d’Accompagnement Psychosociale Groupe Hospitaliser Territorial Val de Marne-Est Saint-Maurice France; 15 17 rue des marronniers Paris France; 16 Department of Psychiatry Hôpital Saint-Antoine Sorbonne Université Paris France; 17 Cabinet de psychologie Bureaux du Polygone Montpellier France; 18 EDT- 194 Bureau de la Colline Saint-Cloud France; 19 CEMKA – 43, boulevard du Maréchal Joffre Bourg-La-Reine France; 20 DueL Nice France

**Keywords:** prospective acceptability, digital health, depression, e-mental health, deprexis, psychotherapy

## Abstract

**Background:**

Major depressive disorder is one of the leading causes of disability worldwide. Although most international guidelines recommend psychological and psychosocial interventions as first-line treatment for mild to moderate depression, access remains limited in France due to the limited availability of trained clinicians, high costs for patients in the context of nonreimbursement, and the fear of stigmatization. Therefore, online blended psychological treatment such as Deprexis could improve access to care for people with depression. It has several advantages, such as easy accessibility and scalability, and it is supported by evidence.

**Objective:**

This study aims to evaluate the real-life acceptability of Deprexis for people with depression in France outside of a reimbursement pathway.

**Methods:**

Deprexis Acceptability Study Measure in Real Life (DARE) was designed as a multicenter cross-sectional study in which Deprexis was offered to any patient meeting the inclusion criteria during the fixed inclusion period (June 2022-March 2023). Inclusion criteria were (1) depression, (2) age between 18 and 65 years, (3) sufficient French language skills, and (4) access to the internet with a device to connect to the Deprexis platform. Exclusion criteria were previous or current diagnoses of bipolar disorder, psychotic symptoms, and suicidal thoughts during the current episode. The primary objective was to measure the prospective acceptability of Deprexis, a new digital therapy. Secondary objectives were to examine differences in acceptability according to patient and clinician characteristics and to identify reasons for refusal. All investigators received video-based training on Deprexis before enrollment to ensure that they all had the same level of information and understanding of the program.

**Results:**

A total of 245 patients were eligible (n=159, 64.9% were women and n=138, 56.3% were single). The mean age was 40.7 (SD 14.1) years. A total of 78% (n=191) of the patients had moderate to severe depression (according to the Patient Health Questionnaire-9 [PHQ-9]). More than half of the population had another psychiatric comorbidity (excluding bipolar disorder, psychotic disorders, and suicidal ideation). A total of 33.9% (n=83) of patients accepted the idea of using Deprexis; the main reason for refusal was financial at 83.3% (n=135). Multivariate logistic regression identified factors that might favor the acceptability of Deprexis. Among these, being a couple, being treated with an antidepressant, or having a low severity level favored the acceptance of Deprexis.

**Conclusions:**

DARE is the first French study aiming at evaluating the prospective acceptability of digital therapy in the treatment of depression. The main reason for the refusal of Deprexis was financial. DARE will allow better identification of factors influencing acceptability in a natural setting. This study highlights the importance of investigating factors that may be associated with the acceptability of digital interventions, such as marital status, medication use, and severity of depression.

## Introduction

Major depressive disorder is a leading cause of disability worldwide, increasing the risk of premature death, reducing the quality of life, and placing a heavy burden on the health care system. It is estimated to affect more than 300 million people worldwide [1]. In its recent report, the World Health Organization (WHO) highlighted that the COVID-19 pandemic, like other ongoing crises, has made the strengthening of mental health systems around the world more urgent than ever [2]. Even before the pandemic, rates of negative lifestyle factors such as physical inactivity, smoking, alcohol consumption, and unhealthy dietary habits were found to be higher in depressed patients and may explain part of the association between depression and mortality [3]. The lockdown may have exacerbated this pattern.

Although international and national guidelines recommend psychological and psychosocial interventions as first-line treatment for mild to moderate depression (since medication alone does not achieve complete remission), these strategies are not properly implemented in France, mainly due to the limited availability of trained clinicians (especially those with cognitive behavioral therapy [CBT] certification) [4].

Over the last 20 years, the field of mental health care for depression has undergone a major technological revolution. Digital health innovations will improve care and could help address some of the key challenges along the depression care pathway. Psychological interventions, such as CBT, are increasingly being delivered over the internet (internet-based CBT) [5], with some interventions being defined as digital therapy.

Digital therapeutics (DTx) deliver evidence-based therapeutic interventions to patients through high-quality software to treat, manage, or prevent a disease or disorder [6]. Nearly a decade ago, they were described by Sepah et al [7] as “evidence-based behavioural treatments delivered online that can increase the accessibility and effectiveness of healthcare.”

Today, personalized treatment selection is entirely possible and necessary to ensure the best allocation of treatment resources for depression [8].

In 2022, National Institute for Health and Care Excellence (NICE) guidelines [9] recommended the use of guided self-help, which includes digitally delivered therapeutic interventions consistent with the concept of DTx, as a first-line treatment. In its 2023 update, the committee conditionally recommended the use of 3 online CBT programs—Beating the Blues, Deprexis, and Space from Depression (Silvercloud) as treatment options for adult depression [10].

Deprexis is a digital psychotherapy program for adult patients enduring major depressive disorder. It is a web-based software solution certified as a class I medical device. It is intended to be used in combination with usual care and has been shown to be effective in 12 randomized controlled trials (N=2901) [11]. Deprexis has been developed on the basis of evidence-based psychological and psychotherapeutic techniques delivered in 10 modules that are available to the patient for 90 days. It also includes exercises and daily activities to support the patient’s progress. In addition, Deprexis can enhance disease and treatment monitoring by integrating a validated assessment questionnaire measuring the intensity of depression symptoms (Patient Health Questionnaire-9 [PHQ-9] [12]), which patients complete on a regular basis. Patients can also share a graphical overview of the evolution of their PHQ-9 scores with their health care professionals. Launched and reimbursed in Germany in 2021, Deprexis was available in the United Kingdom and France in June 2022, in Spain in May, and in Italy in October 2023. A group of experts has highlighted the lack of specific regulation at the European level and the heterogeneity of access as a need for action [13].

**Figure 1:**
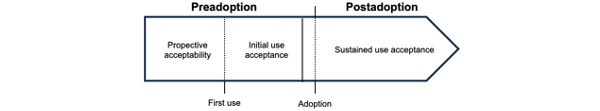
Proposed terminology for technology acceptance lifecycle according to Nadal et al [16].

Even though DTx are now part of the therapeutic options, understanding the factors that determine their uptake in the health care landscape is critical [14]. Acceptability is a core concept in digital health and has not yet reached a consensus definition. Perski and Short [15] proposed a dynamic model linking the concept of acceptability, user engagement, and intervention effectiveness to report acceptability. The authors noted that the perceptions of acceptability can be formed after learning about a new intervention but before engaging with it, which they referred to as “preuse acceptability” or “prospective acceptability.” In a recent publication, Nadal et al [16] proposed an evolving terminology according to a life cycle (Figure 1). It implicitly includes the notions of “constraints” and “facilities” of use. In medicine, several dimensions may contribute to the acceptability of new technology, including the cognitive aspects involved and the cultural framework in which it is situated (ie, professional culture), as well as organizational, social, and ergonomic aspects, in addition to its centrality to medicine and the balance of benefits and risks. When proposing a composite model of acceptability in the technical domain, more specific dimensions are encountered, such as usability, reliability, use, and risk.

Therefore, the first objective of the Deprexis Acceptability Study Measure in Real Life (DARE) study was to explore the prospective acceptability of a digital therapy such as Deprexis outside the “classical reimbursement pathway” in France.

Secondary objectives were defined to explore potential differences in prospective acceptability according to setting (private or public) and practitioner profile (type and setting of practice), patient severity (assessed with PHQ 9), and ancillary treatment (including psychotherapy). It was also important to analyze the reasons for the refusal of Deprexis and the refusal rate over time.

## Methods

### Study Design

DARE has been designed as a cross-sectional, multicenter, naturalistic study in which Deprexis will be offered to any patient who meets the inclusion criteria (taking into account the exclusion criteria) during the fixed inclusion period from June 2022 to March 2023.

### Participant Recruitment

Patients were recruited by 12 volunteer investigators (psychiatrists and psychologists) working in a sample of centers identified by an expert panel. All investigators had access to video-based training on Deprexis prior to enrollment to ensure that they all had the same level of information and understanding of Deprexis. They also received a study presentation and a patient information or nonobjection letter (General Data Protection Regulation) together with a leaflet explaining Deprexis. They were able to call other members of their team (nurse, psychiatrist, and physician) to help with the recruitment. All patients with a depressive disorder, regardless of severity, who were older than 18 years of age and younger than 65 years of age were eligible. They were also required to be fluent in French and have internet access with a device to connect to the Deprexis platform. According to the European conformity mark indication of Deprexis, patients were excluded if they were diagnosed with bipolar disorder; psychotic disorders, such as schizophrenia; or if they had suicidal ideation in the current episode.

### Ethical Considerations

All participants received detailed information about the study and were not requested to provide consent as stipulated in the Reference Methodology (MR-004). The MR-004 is a procedure developed by the French Data Protection Authority (Commission nationale de l'informatique et des libertés; CNIL) to simplify health data processing without the need to obtain patient consent for every study, which exempts it from an institutional review board approval. We have appended the declaration of compliance with the MR-004 reference methodology (Multimedia Appendix 1). An agreement was signed to ensure compliance with the CNIL’s policy on patient consent.

Statements regarding the anonymity of responses or confidentiality of data were maintained throughout the study. No compensation or reward was offered to participants in this study.

### Data Collection

Data were collected in an electronic case report form (Multimedia Appendix 2) and stored in a certified health data hosting facility. The questionnaire was completed by the investigator for all patients included in the study, regardless of whether they agreed to use Deprexis or not. It consisted of two parts, (1) investigator characteristics and (2) patient information including sociodemographic characteristics, clinical characteristics, and acceptance of the system.

### Statistical Analysis

Statistical analysis was performed using SAS software (SAS Institute). Both descriptive and comparative analyses were performed. The first was used to describe investigator and patient characteristics. The second was performed to compare the patient profile according to acceptance of Deprexis and the reason for refusal. Multivariate logistic analysis was used to identify factors that could explain acceptance. Explanatory factors included in the model were sociodemographic and clinical characteristics of the patient and the profession of the practitioner who admitted the patient. Correlation analysis and bivariate models were performed to ensure convergence of the multivariate model. A stepwise method, with an alpha risk of 20% to enter and remain in the model, was used to automatically select criteria in the final model. A sensitivity analysis was performed by including all criteria in the logistic model.

## Results

### Patients’ Recruitment

Of the 15 investigators who volunteered to participate, 12 participants, including 10 psychiatrists and 2 psychologists, enrolled patients. The different practice contexts were represented, with 5 investigators working in private practice and 7 in public practice (hospital or community-based centers).

Between June 2022 and March 2023, 248 patients were enrolled, of whom 245 were eligible. Enrollment was relatively stable during the enrollment period. Most patients (n=222, 90.6%) were enrolled by a psychiatrist as principal investigator or their team—mainly 50.7% (n=124) by a psychiatrist, 22.4% (n=55) by another team doctor, and 17.1% (n=42) by a team nurse. Only 9.4% (n=23) of patients were seen by a psychologist. Overall, 54.7% (n=134) of patients were seen in public consultations, 30.2% (n=74) in private consultations, and 12.2% (n=30) in-hospital consultations. Investigators included between 7 and 60 patients.

### Patient Characteristics

Of the 245 eligible patients, 159 (64.9%) were female and 138 (56.3%) were single. The mean age was 40.7 (SD 14.1) years. At the time of the study, 99 (40.4%) were employed, 52 (21.2%) were on sick leave, 41 (16.7%) were unemployed, 36 (14.7%) were students, and 17 (6.9%) were retired (Table 1).

At enrollment, more than three-quarters of patients (n=191, 78%) had moderate to severe depression (according to PHQ-9) and a quarter of patients had at least 1 depressive episode in their history (n=127, 51.8% between 1 and 3 episodes, n=58, 23.7% more than 3 episodes). In 21.6% (n=53) of patients, the use of Deprexis was suggested for the first episode of depression. More than half of the population (n=135, 55.1%) had another psychiatric comorbidity, excluding bipolar disorder, psychotic disorders, and suicidal ideation. Among patients with another psychiatric disorder, 50.4% (n=68) had an anxiety disorder, 22.2% (n=30) had attention-deficit/hyperactivity disorder, and 17.0% (n=23) had a personality disorder. A small number of patients had alcohol and drug use or eating disorders (n=10, 7.5%). More than 80% (n=195) of the patients were treated with antidepressants (n=184, 76.0%), anxiolytics (n=101, 41.9%), or psychotherapy (n=59, 24.6%) for the current depressive episode.

**Table 1:** Sociodemographic and clinical characteristics of eligible patients (n=245).

Characteristics			Eligible patients (n=245)
**Sociodemographic characteristics**			
	Women, n (%)		159 (64.9)
	Age in 2022 (years), mean (SD)		40.7 (14.1)
	**Marital status (MD^a^=1), n (%)**		
		Single	138 (56.3)
		Couple	106 (43.3)
	**Professional status (current), n (%)**		
		Professional	99 (40.4)
		On sick leave	52 (21.2)
		Unemployed	41 (16.7)
		Student	36 (14.7)
		Retired	17 (6.9)
	**Financial resources, n (%)**		
		Paid profession	142 (58.0)
		Without resources	31 (12.7)
		Unemployed or minimum state help	28 (11.4)
		Disability pension	23 (9.4)
		Retirement pension	16 (6.5)
		Without specific information related to their resources	5 (2.0)
**Clinical characteristics**			
	**Severity of depressive symptoms (PHQ-9^b^ score), n (%)**		
		1-4: little	14 (5.7)
		5-9: mild	40 (16.3)
		10-14: moderate	69 (28.2)
		15-19: moderately severe	71 (29.0)
		≥20: severe	51 (20.8)
	**History of depressive episodes, n (%)**		
		None	53 (21.6)
		1-3 depressive episodes	127 (51.8)
		>3 depressive episodes	58 (23.7)
		Unknown	7 (2.9)
	At least 1 other psychiatric comorbidity, n (%)		135 (55.1)
	Current antidepressant, anxiolytic, or psychotherapy, n (%)		199 (82.2)
	Current antidepressant or anxiolytic, n (%)		195 (80.6)

^a^MD: missing data.

^b^PHQ-9: Patient Health Questionnaire-9 (Multimedia Appendix 3).

### Prospective Acceptability

A total of 33.9% (n=83) of patients accepted the idea of using Deprexis. Participants could give several reasons for refusal. The main reason for refusal was financial (n=135, 83.3% of patients). A total of 54 (33.3%) patients refused because of the digital tool and 32 (19.9%) for another reason. These reasons included the fact that they were already receiving treatment for depression, loneliness, and lack of interest in any kind of therapy.

Of the patients who refused Deprexis only for financial reasons (n=135, 83.3%), 71.9% (n=97) were women. This proportion was statistically higher (*P*=.02) than among patients who accepted the principle of the device (n=49, 59.0%) or those who refused for at least 1 reason other than financial (n=13, 48.1%). More individual patients were identified in this subgroup (n=92, 68.1% vs n=34, 41.0% in the Deprexis-accepted group and n=12, 44.4% in the Deprexis refused for other reasons group, *P*<.001). A lower rate of participants treated with anxiolytics or antidepressants was observed (n=97, 72.9% vs n=72, 87.8% and n=26, 96.3%, respectively, *P*=.003). This difference was observed for the 2 treatments separately and was statistically significant for antidepressants (n=92, 69.2% vs n=68, 82.9% and n=24, 88.9%).

**Figure 2:**
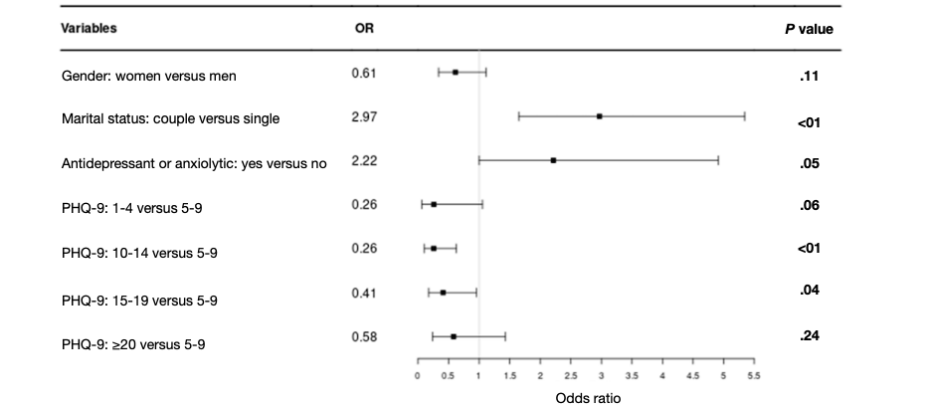
Results of the multivariate logistic regression explaining acceptation of Deprexis. OR: odds ratio; PHQ-9: Patient Health Questionnaire-9.

Multivariate logistic regression was performed to identify factors that might favor the acceptability of Deprexis, using an automatic selection of characteristics as described. Figure 2 shows the results of this logistic regression. Patients in couples were 3 times more likely to accept the device (odds ratio [OR] 2.97, 95% CI 1.65-5.34; *P*<.001), and patients treated with antidepressants or anxiolytics were twice as likely to accept the device (OR 2.22, 95% CI 1.00-4.91; *P*=.049). The severity of depression had a statistically significant negative effect on acceptability (*P*=.04): patients with moderate depression (PHQ-9 range 10-14) were 2.4 times more likely to refuse the device (OR 0.26, 95% CI 0.10-0.63; *P*=.003) than patients with mild depression. This rate is 3.8 for patients with moderate depression (PHQ-9 range 15-20; OR 0.41, 95% CI 0.18-0.96; *P*=.04). Gender did not have a significant effect. A sensitivity analysis was performed including all characteristics in the multivariate model. The results were similar to those presented above. It showed that age, gender, professional status, resources, psychiatric history, or comorbid psychiatric disorders had no effect on acceptability.

## Discussion

### Principal Findings

DARE is the first French study to assess the prospective acceptability of digital therapy in the treatment of depression. About one-third of the patients accepted the idea of using Deprexis. The main reason given by patients for not using Deprexis was financial, especially among women. DARE allows better identification of factors influencing acceptability, such as marital status (patients in couples were 3 times more likely to accept the device) or medication use (patients treated with antidepressants and anxiolytics were twice as likely to accept the device) in a natural setting. Finally, the severity of depression was negatively correlated with acceptability.

Many patients experiencing depression refuse appropriate treatment, and there is a tendency for the treatment to be underused due to barriers to access and long waiting times. For those who do accept treatment, it can be difficult to achieve remission despite treatment according to international recommendations. To optimize the treatment of depression, digital tools have been developed and are supported by evidence [7]. Deprexis has been shown to be effective in 12 randomized controlled trials (N=2901) [11]. Since the publication of this meta-analysis, other studies have provided additional support for Deprexis. The program has been shown to be effective in other sociocultural contexts [17]; however, since January 2024, the program is no longer available in France, Spain, the United Kingdom, or Italy, which will limit its reach; it improves functioning in daily life compared with usual treatment alone in patients previously treated for major depressive episodes in hospital [18]; in Germany, a 12-week course of Deprexis treatment results in lower care costs [19].

The main barriers to accessing psychotherapy are the availability of a therapist and reimbursement. The DTx can address the barrier of limited access due to the limited number of trained health professionals, but it raises questions about the acceptance of this type of tool by the French population and, more specifically, its acceptability when prescribed by a health professional without reimbursement. In our study, the main reason for refusal was financial. In a survey of patients with depression [20], the fact that all costs are covered is a factor that facilitates acceptance. Our results may differ from those in other countries, such as Germany or the United States, where Deprexis is reimbursed when prescribed by a health care professional. However, it raises the issue of patients’ ability to make their own decisions about spending money on their health. In addition, it highlights the fact that reimbursement decisions by health authorities can have an impact on the use or nonuse of health care by patients enduring depression. Women were more likely than men to refuse treatment for financial reasons, raising questions about the impact of gender differences on depression [21]. A recent meta-analysis showed that women were less likely to participate in this type of program [22]. The relationship between income class and inequality and gender differences in depression is particularly complex given the variety of measures of gender equality available. Financial inequalities between the male and female genders may be a factor in women’s nonuse of health care, putting them at a disadvantage when they do need treatment. Being single for a woman is also associated with financial refusal in our study, suggesting that social insecurity should be considered as an additional factor in refusal.

In line with the previous hypothesis, our study shows that marital status is strongly associated with the prediction of acceptability—being a couple multiplies the propensity to accept Deprexis by a factor of 3. Not living alone can reduce the financial burden of nonreimbursement in France. Similarly, adherence to medication was strongly associated with the acceptability of Deprexis in our study. People who are adhering to traditional care may, therefore, be more likely to accept additional programs to treat their depression. It also shows that patients who refuse treatment are the least likely to access complementary therapies, which calls for the development of tools for health care professionals to increase overall adherence to treatment for people with depression. Specifically, the number and intensity of depressive symptoms (loss of hope, having no motivation or interest in things, cognitive dysfunction, feeling guilt-ridden, etc) may hinder the process of treatment adherence. Patients may find it difficult to believe that a digital therapeutic program could really be helpful, perhaps, when they feel seriously depressed. This pessimistic appraisal may also reflect the typical depressive negativity (ie, the negative “cognitive triad”) [23]. Our results point in this direction, as patients whose depression was classified as moderate were less likely to accept Deprexis than patients whose depression was classified as mild. The clinician is caught in a vicious circle in which the positive evolution of a patient’s symptoms depends on the complementary tools they find in the program, the acceptability of which depends on the level of clinical severity. However, Deprexis has been shown to be effective, even in people with severe symptoms [24]. Health care professionals should, therefore, question the way they present care to optimize adherence to prescribed treatments. Although every effort should be made at the earliest stages of depression, the situation of the French health care system (eg, lack of nursing staff and nonreimbursement) makes this an illusory prospect. On the other hand, a graduated approach to the intensity of care according to the severity of symptoms is essential. Professionals trained in motivational interviewing could specifically support patients with severe symptoms in their treatment, encouraging patients to consider that achieving their life goals can be supported by therapeutic tools such as Deprexis. Cognitive restructuring, used in the field of cognitive and behavioral therapies, can also help to soften patterns of failure or incurability in patients experiencing depression and promote the acceptance of treatment. Finally, this study did not show any differences in acceptability according to age. This result is rather counterintuitive but suggests that age, especially older age, should not be seen as an obstacle to the implementation of digital therapy. In fact, older adults are generally positive about internet-based interventions [25].

In this study, professionals presented Deprexis to people experiencing depression after explaining how the program worked and what they could expect to gain from it. We measured prospective acceptability according to the method described by Perski and Short [15]. Although all investigators received the same video-based training on Deprexis prior to enrollment, we cannot ascertain why it was more difficult in some encounters than others to persuade the patients to use the program. In our study, the program was mainly presented by psychiatrists or their teams (rather than psychologists), in both public and private practice. DTx is underdeveloped in psychiatry in France, and our results call into question the ability of psychologists to use this type of tool, as those who participated in our study did not recruit enough participants. Before attempting to motivate patients to use these programs, health care professionals need to be convinced of their usefulness to ensure their commitment [26]. Deprexis is based on a variety of psychotherapeutic techniques, including CBT. A limited number of health care professionals in France are certified to deliver this type of psychotherapy, even though it is at the forefront of international treatment recommendations, particularly, for depression. A major effort needs to be made to ensure that psychologists in France are trained in these approaches and are aware of the value of programs such as Deprexis.

### Limitations

This trial has several limitations. First, the number of centers was small because of difficulties in recruiting investigators. These difficulties could be related to the reluctance of professionals to present nonreimbursed therapies to their patients, a rejection, or at least a hesitation to suggest the use of digital therapies as a complement to usual care. As a reminder, similar programs are not reimbursed in France and are rarely used. The investigators who agreed to recruit patients did so on a voluntary basis. Therefore, potential bias in patient recruitment cannot be ruled out. We can also assume that the investigators were at least not resistant to digital tools, which may have influenced their communication with patients. Another limitation is the lack of data on the effective use of Deprexis as this study only considered “prospective acceptability.” We were, therefore, not able to assess the “real-world” acceptance of Deprexis, which according to Nadal et al [16] is associated with perceived usefulness, intention to use, actual use, user satisfaction, perceived ease of use, attitude toward use, system usability, system quality, user feedback, and user enjoyment. In the future, studies evaluating “prospective acceptability” and “real-world acceptance” for the same patients should be carried out. Acceptance assessors should not be those who carried out the acceptability assessment, or at least should be blind to the results of that assessment. Finally, the patients were recruited during a visit or routine follow-up with psychiatrists or psychologists, and it would have been good to involve general practitioners to have a better representation of the general population of patients with depression. It is not possible for us to specify the financial reasons as to why patients in the study refused to take Deprexis. In fact, the reasons for refusal in the questionnaire available to the investigators were collected by means of a simple open question asking patients to specify their reasons for refusal.

### Conclusions

DARE is the first study with Deprexis to acknowledge the influence of several factors on the acceptability of digital intervention, such as marital status and the use of antidepressant or anxiolytic medication. As noted by van Gemert-Pijnen et al [27], human characteristics, the socioeconomic and cultural environment, and technologies are closely linked. Taking these different factors into account in a more holistic approach could improve the uptake and impact of eHealth technologies.

## Appendix

Multimedia Appendix 1 Declaration of compliance with the MR-004 reference methodology (2226552).

Multimedia Appendix 2 Deprexis Acceptability Study Measure in Real Life (DARE) questionnaire (020622 VF).

Multimedia Appendix 3 Patient Health Questionnaire-9 (VF).

## Abbreviations

CBT: cognitive behavioral therapy
CNIL: Commission nationale de l'informatique et des libertés
DARE: Deprexis Acceptability Study Measure in Real Life
DTx: digital therapeutic
NICE: National Institute for Health and Care Excellence
PHQ-9: Patient Health Questionnaire-9
WHO: World Health Organization

## Data Availability

The data sets used for this study are available from the corresponding author upon reasonable request.

## References

[1] Arias-de la Torre J, Vilagut G, Ronaldson A, Serrano-Blanco A, Martín V, Peters M, Valderas JM, Dregan A, Alonso J (2021). Prevalence and variability of current depressive disorder in 27 European countries: a population-based study. Lancet Public Health.

[2] The impact of COVID-19 on mental health cannot be made light of. World Health Organization.

[3] Cuijpers P, Schoevers RA (2004). Increased mortality in depressive disorders: a review. Curr Psychiatry Rep.

[4] Rapport sur la qualification d’une approche population centrée et partenariale en santé mentale. France Stratégie, République Française.

[5] Andersson G, Titov N, Dear BF, Rozental A, Carlbring P (2019). Internet-delivered psychological treatments: from innovation to implementation. World Psychiatry.

[6] Digital Trade Alliance.

[7] Sepah SC, Jiang L, Peters AL (2015). Long-term outcomes of a web-based diabetes prevention program: 2-year results of a single-arm longitudinal study. J Med Internet Res.

[8] Karyotaki E, Efthimiou O, Miguel C, Bermpohl FMG, Furukawa TA, Cuijpers P, Riper H, Patel V, Mira A, Gemmil AW, Yeung AS, Lange A, Williams AD, Mackinnon A, Geraedts A, van Straten A, Meyer B, Björkelund C, Knaevelsrud C, Beevers CG, Botella C, Strunk DR, Mohr DC, Ebert DD, Kessler D, Richards D, Littlewood E, Forsell E, Feng F, Wang F, Andersson G, Hadjistavropoulos H, Christensen H, Ezawa ID, Choi I, Rosso IM, Klein JP, Shumake J, Garcia-Campayo J, Milgrom J, Smith J, Montero-Marin J, Newby JM, Bretón-López J, Schneider J, Vernmark K, Bücker L, Sheeber LB, Warmerdam L, Farrer L, Heinrich M, Huibers MJH, Kivi M, Kraepelien M, Forand NR, Pugh N, Lindefors N, Lintvedt O, Zagorscak P, Carlbring P, Phillips R, Johansson R, Kessler RC, Brabyn S, Perini S, Rauch SL, Gilbody S, Moritz S, Berger T, Pop V, Kaldo V, Spek V, Forsell Y, Individual Patient Data Meta-Analyses for Depression (IPDMA-DE) Collaboration (2021). Internet-based cognitive behavioral therapy for depression: a systematic review and individual patient data network meta-analysis. JAMA Psychiatry.

[9] (2022). Depression in adults: treatment and management. National Institute for Health and Care Excellence (NICE).

[10] (2023). Digitally enabled therapies for adults with depression: early value assessment. National Institute for Health and Care Excellence (NICE).

[11] Twomey C, O'Reilly G, Bültmann O, Meyer B (2020). Effectiveness of a tailored, integrative internet intervention (deprexis) for depression: updated meta-analysis. PLoS One.

[12] Kroenke K, Spitzer RL, Williams JBW (2001). The PHQ-9: validity of a brief depression severity measure. J Gen Intern Med.

[13] Courtet P, Amiot O, Baca-Garcia E, Bellardita L, Cerveri G, Clair AH, De Leo D, Drapier D, Fakra E, Gheysen F, Giner L, Gonzalez-Pinto A, Gussoni G, Haffen E, Lecardeur L, Mayoral-Cleries F, Mennini FS, Sáiz PA, Vieta E, Hidalgo DA, Volpe U (2023). Understanding the potential of digital therapies in implementing the standard of care for depression in Europe. Eur Psychiatry.

[14] van Kessel R, Roman-Urrestarazu A, Anderson M, Kyriopoulos I, Field S, Monti G, Reed SD, Pavlova M, Wharton G, Mossialos E (2023). Mapping factors that affect the uptake of digital therapeutics within health systems: scoping review. J Med Internet Res.

[15] Perski Olga, Short CE (2021). Acceptability of digital health interventions: embracing the complexity. Transl Behav Med.

[16] Nadal C, Sas C, Doherty G (2020). Technology acceptance in mobile health: scoping review of definitions, models, and measurement. J Med Internet Res.

[17] Lopes RT, da Rocha GC, Svacina MA, Meyer B, Šipka D, Berger T (2023). Effectiveness of an internet-based self-guided program to treat depression in a sample of Brazilian users: randomized controlled trial. JMIR Form Res.

[18] Richter LE, Machleit-Ebner A, Scherbaum N, Bonnet U (2023). How effective is a web-based mental health intervention (deprexis) in the treatment of moderate and major depressive disorders when started during routine psychiatric inpatient treatment as an adjunct therapy? A pragmatic parallel-group randomized controlled trial. Fortschr Neurol Psychiatr.

[19] Gräfe V, Moritz S, Greiner W (2020). Health economic evaluation of an internet intervention for depression (deprexis), a randomized controlled trial. Health Econ Rev.

[20] Hafner J, Schönfeld S, Tokgöz P, Choroschun K, Schlubach A, Dockweiler C (2022). Digital health interventions in depression care-a survey on acceptance from the perspective of patients, their relatives and health professionals. Healthcare (Basel).

[21] Salk RH, Hyde JS, Abramson LY (2017). Gender differences in depression in representative national samples: meta-analyses of diagnoses and symptoms. Psychol Bull.

[22] Borghouts J, Eikey E, Mark G, De Leon C, Schueller SM, Schneider M, Stadnick N, Zheng K, Mukamel D, Sorkin DH (2021). Barriers to and facilitators of user engagement with digital mental health interventions: systematic review. J Med Internet Res.

[23] Moritz S, Schröder J, Meyer B, Hauschildt M (2013). The more it is needed, the less it is wanted: attitudes toward face-to-face intervention among depressed patients undergoing online treatment. Depress Anxiety.

[24] Meyer B, Bierbrodt J, Schröder J, Berger T, Beevers CG, Weiss M, Jacob G, Späth C, Andersson G, Lutz W, Hautzinger M, Löwe B, Rose M, Hohagen F, Caspar F, Greiner W, Moritz S, Klein JP (2015). Effects of an internet intervention (deprexis) on severe depression symptoms: randomized controlled trial. Internet Interv.

[25] McMurchie W, Macleod F, Power K, Laidlaw K, Prentice N (2013). Computerised cognitive behavioural therapy for depression and anxiety with older people: a pilot study to examine patient acceptability and treatment outcome. Int J Geriatr Psychiatry.

[26] Bourla A, Ferreri F, Ogorzelec L, Peretti CS, Guinchard C, Mouchabac S (2018). Psychiatrists' attitudes toward disruptive new technologies: mixed-methods study. JMIR Ment Health.

[27] van Gemert-Pijnen JEWC, Nijland N, van Limburg M, Ossebaard HC, Kelders SM, Eysenbach G, Seydel ER (2011). A holistic framework to improve the uptake and impact of eHealth technologies. J Med Internet Res.

