# Supplementary material for: Prospective Acceptability of Digital Therapy for Major Depressive Disorder in France: Multicentric Real-Life Study
Source: JMIR Form Res. 2024 May 20;8:e53204. doi: 10.2196/53204 (PMC11148517; doi:10.2196/53204)
Supplement: Multimedia Appendix 1 [file formative_v8i1e53204_app1.pdf]

Référence CNIL :

2226552 v 0

Déclaration de conformité

au référentiel de méthodologie de référence MR-004

reçue le 1 juin 2022

Madame Hélène MOORE
ETHYPHARM DIGITAL THERAPY
DIRECTION GÉNÉRALE
194 BUREAUX DE LA COLLINE
92213 ST CLOUD CEDEX

ORGANISME DÉCLARANT

Nom : ETHYPHARM DIGITAL THERAPY

Service :

Adresse : 194 BUREAUX DE LA COLLINE

CP : 92213

Ville : ST CLOUD CEDEX

N° SIREN/SIRET : 879453298 00017

Code NAF ou APE : 7740Z

Tél. :

Fax. :

Par la présente déclaration, le déclarant atteste de la conformité de son/ses traitement(s) de données à caractère personnel au référentiel mentionné ci-dessus.

La CNIL peut à tout moment vérifier, par courrier ou par la voie d'un contrôle sur place ou en ligne, la conformité de ce(s) traitement(s).

Fait à Paris, le 2 juin 2022

Les données personnelles nécessaires à l'accomplissement des missions de la CNIL sont conservées et traitées dans des fichiers destinés à son usage exclusif. Les personnes concernées peuvent exercer leurs droits Informatique et Libertés en s'adressant au délégué à la protection des données de la CNIL via un formulaire en ligne ou par courrier postal.

Pour en savoir plus : https://www.cnil.fr/donnees-personnelles
