# Supplementary material for: Prospective Acceptability of Digital Therapy for Major Depressive Disorder in France: Multicentric Real-Life Study
Source: JMIR Form Res. 2024 May 20;8:e53204. doi: 10.2196/53204 (PMC11148517; doi:10.2196/53204)
Supplement: Multimedia Appendix 2 [file formative_v8i1e53204_app2.docx]

Etude DARE

Deprexis® étude exploratoire d’Acceptabilité mesurée en vie **RE**elle

Questionnaire investigateur (à compléter une seule fois lors de l’acceptation de l’étude)

**Genre**

1. Masculin
2. Féminin
3. Non-binaire

**Année de naissance** : /__/__/__/__/

**Profil** :

1. Psychiatre
2. Psychologue
3. Infirmier
4. Autre, préciser : __________________________________

**Département d’exercice :** /__/__/

**Mode d’exercice***(plusieurs réponses possibles)*

_1_ Consultations publiques (CMP, ou autres)

_1_ Consultations privées (cabinet libéral ou autres)

_1_ Unité d’hospitalisation publique

_1_ Unité d’hospitalisation en clinique privée

_1_ Autre, précisez : _______________________________________

Questionnaire à compléter pour chaque patient à qui deprexis® est proposé

Code anonymisé centre : (pré-renseigné dans le eCRF)

Code anonymisé investigateur : (pré-renseigné dans le eCRF)

Code anonymisé patient : (pré-renseigné dans le eCRF, s’incrémente)

Date de proposition de deprexis® : /__/__/ /__/__/ /__/__/__/__/

Patient correspondant aux critères d’inclusion (EDC toute sévérité, entre 18 ans et 65 ans, maîtrise suffisante du français et accès à internet et un média de connexion) :

1. Oui
2. Non -> Arrêt du questionnaire (patient non éligible à deprexis®)

Patient présentant au moins un critère d’exclusion (troubles bipolaires, maladies psychotiques, idées suicidaires au cours de l’épisode actuel)

1. Oui -> Arrêt du questionnaire (patient non éligible à deprexis®)
2. Non

| **Genre**   1. Masculin 2. Féminin 3. Non-binaire   **Année de naissance** /__/__/__/__/  **Statut matrimonial**   1. Célibataire 2. En couple (marié, pacsé, concubinage) 3. Non connu   **Enfants**   1. Oui 2. Non 3. Non connu   **Statut professionnel**   1. Sans emploi 2. Etudiant 3. Activité professionnelle 4. Actuellement en arrêt de travail 5. Retraité (e) 6. Non connu   **Ressources**   1. Sans ressource 2. Activité professionnelle 3. Chômage, RSA 4. Pension d’invalidité, AAH (MDPH) 5. Retraite 6. Non connues   **Lieu de prescription de deprexis®**   1. Consultations publiques (CMP, ou autres) 2. Consultations privées (Cabinet libéral ou autres) 3. Unité d’hospitalisation publique 4. Unité d’hospitalisation en clinique privée 5. Autres | **Antécédents psychiatriques**   1. Aucun 2. 1-3 épisode(s) dépressif(s) caractérisé(s) 3. > 3 épisodes dépressifs caractérisés 4. Autres antécédents psychiatriques (précisez __________________________________) 5. Non connu   **Comorbidité(s) psychiatrique(s) actuelle(s)**  Précisez : __________________________________  **Traitement en cours** (plusieurs réponses possibles)   1. Aucun 2. Antidépresseur 3. Anxiolytique 4. Psychothérapie 5. Non connu   **Sévérité de la dépression : score total au PHQ9** /__/__/  **Accepte le dispositif deprexis®**   1. Oui 2. Non   **Si non, raison(s) de refus de deprexis®** (plusieurs réponses possibles)   1. Financière 2. Technologie numérique 3. Autres, précisez : ________________________   Précisez si besoin : _____________________________  ___________________________________________  ___________________________________________ |
| --- | --- |
