# Supplementary material for: Prospective Acceptability of Digital Therapy for Major Depressive Disorder in France: Multicentric Real-Life Study
Source: JMIR Form Res. 2024 May 20;8:e53204. doi: 10.2196/53204 (PMC11148517; doi:10.2196/53204)
Supplement: Multimedia Appendix 3 [file formative_v8i1e53204_app3.docx]

Etude DARE - Autoquestionnaire patient Num patient : /__/__/

**Au cours des 2 dernières semaines, selon quelle fréquence avez-vous été gêné(e) par les problèmes suivants ?**

|  | Jamais | Plusieurs jours | Plus de la moitié du temps | Presque tous les jours |
| --- | --- | --- | --- | --- |
| 1. Peu d’intérêt ou de plaisir à faire les choses | _0_ | _1_ | _2_ | _3_ |
| 1. Être triste, déprimé(e) ou désespéré(e) | _0_ | _1_ | _2_ | _3_ |
| 1. Difficultés à s’endormir ou à rester endormi(e), ou dormir trop | _0_ | _1_ | _2_ | _3_ |
| 1. Se sentir fatigué(e) ou manquer d’énergie | _0_ | _1_ | _2_ | _3_ |
| 1. Avoir peu d’appétit ou manger trop | _0_ | _1_ | _2_ | _3_ |
| 1. Avoir une mauvaise opinion de soi-même, ou avoir le sentiment d’être nul(le), ou d’avoir déçu sa famille ou s’être déçu(e) soi-même | _0_ | _1_ | _2_ | _3_ |
| 1. Avoir du mal à se concentrer, par exemple, pour lire le journal ou regarder la télévision | _0_ | _1_ | _2_ | _3_ |
| 1. Bouger ou parler si lentement que les autres auraient pu le remarquer. Ou au contraire, être si agité(e) que vous avez eu du mal à tenir en place par rapport à d’habitude | _0_ | _1_ | _2_ | _3_ |
| 1. Penser qu’il vaudrait mieux mourir ou envisager de vous faire du mal d’une manière ou d’une autre | _0_ | _1_ | _2_ | _3_ |

Score total : /__/__/ (à noter par le professionnel de santé et à reporter dans le eCRF)

Calculer le score total en sommant les chiffres associés aux 9 réponses et reporter ce score dans l’eCRF
